# Breast Cancer Awareness among Female School Teachers in Saudi Arabia: A Population Based Survey

**DOI:** 10.31557/APJCP.2020.21.2.337

**Published:** 2020

**Authors:** Basem Alshareef, Waed Yaseen, Wed Jawa, Yasmeen Barnawe, Wejdan Alshehry, Heba Alqethami, Wala Bukari, Osama Alqumaili

**Affiliations:** 1 *Consultant General Surgery, Department of General Surgery, Alnoor Specialized Hospital, *; 2 *Department of Obstetric and Gynecology, Maternity and Children’s Hospital, *; 5 *Department of General Surgery, King Faisal General Hospital, *; 6 *Department of Obstetric and Gynecology, Heraa General Hospital, Makkah, *; 3 *Resident Community Medicine, *; 4 *Department of Obstetric and Gynecology, Armed Forces Hospital, Taif, Saudi Arabia. *

**Keywords:** Breast cancer, Saudi Arabia, screening, awareness, knowledge, school teachers

## Abstract

**Introduction:**

Breast cancer (BC) is the most frequent malignancy in women and second leading cause of cancer related death worldwide. In Saudi Arabia, it is the ninth leading cause of death. Few studies have been conducted to address BC awareness in KSA in general and to our knowledge, this is the first to be conducted in the Makkah region.

**Aim:**

To assess the level of awareness, knowledge and attitude of Saudi female teachers towards BC, in primary intermediate and secondary schools within the Makkah region.

**Method and Materials:**

The study proposal was approved by the Research Ethical Committee in the faculty of medicine, at Umm Al-Qura University. A self-administered questionnaire on BC was designed and tested. The questionnaire consisted of 23 items and covered four domains (awareness about the etiology, knowledge about BC risk factors, symptoms, knowledge about diagnosis and treatment& attitude toward screening). A sample of 400 female school teachers (working in primary, intermediate and secondary schools) were selected by multistage, random sampling. A selection of forty schools, with a sample of 10 teachers from each, was chosen at random in Makkah. Proper permission was obtained from the authorities. the questionnaire was filled out by each participant. The collected data was statistically analyzed using SPSS version 21.

**Results:**

The results showed that knowledge and attitude about BC amongst the female teachers differed significantly by age and marital status. Those aged 46-55 (F=8.5, p>0.002) and those who are married (F=2.7, p>0.04) had more knowledge about BC than others. The majority of respondents had a limited level of knowledge and understanding of BC symptoms (Table 2). However, it also showed that the teachers are very enthusiastic to learn about BC, and its prevention. Most participants (40/%) reported that they had not performed any breast exams before.

**Conclusions and Recommendation:**

This study indicated that Saudi female teachers’ level of knowledge of BC is inadequate. Introducing and developing an effective health education program in female schools within KSA is recommended.

## Introduction

Brest Cancer (BC) as a multifactorial disease is the most common cancer and the second leading cause of deaths among women worldwide. The incidence of BC is rising more rapidly in the population group that used to experience low incidence of the disease and it reduces the life expectancy of the population at risk (especially those between 31 and 50) (Adebamowo and Adekunle, 1999).

Global statistics show that the annual morbidity and mortality of BC is increasing Over1.15 million women worldwide (representing 10% of all diagnosed cancers and 23% of cancers diagnosed in women) are diagnosed with BC each year and more than 502,000 of them die from the disease (more than 1.6% of all female mortality worldwide) (Ferlay et al., 2007).

Women in the Middle East face a significant risk of high mortality rate from BC, due to the delay in the diagnosis and the advanced stages of the disease at the time of diagnosis (Alharbi et al., 2012; Abulkhair et al., 2010). In kingdom of Saudi Arabia, BC is usually diagnosed at late stage and more frequently in young, pre-menopausal women, under the age of 45, in comparison to western countries (Alharbi et al., 2012; Abulkhair et al., 2010).

The etiologies of the majority of BCs are unknown, with only about 25% to 40% of them attributed to well known risk factors (ACS, 2009).

Awareness of BC risk factors (gender, age, family, personal history, racial factors, radiation exposure, breast changes, early menarche, late menopause, prolonged null parity, obesity, diet, alcohol consumption, tobacco smoking, excessive estrogen exposure, oral contraceptive use, stress, anxiety) and perception of personal risk, are important factors for motivation, prevention, and/or early detection of the disease (Bassey et al., 2010; Irurhe et al., 2011; Rojas and Stuckey, 2011). Knowledge about the disease (including screening methods and warning signs) plays an important and effective role towards developing and employing screening programs in a community. This can effectively improve the chances of early detection of BC, should result in an improvement in survival rates and quality of life (Elmore et al., 2005).

Late diagnosis of BC is mainly due to lack of awareness in the population and barriers to access health services (Abulkhair et al., 2010; WHO, 2012).

Early detection of BC can be achieved through: Implementing effective screening programs and annual mammogram in the targeted population; Improving public awareness about signs and symptom of BC; and the encouragement of females to take a prompt action (Abulkhair et al., 2010; Alam, 2006; Burgess et al., 2009; Forbesljl et al., 2011) Unfortunately, in Saudi Arabia, we don’t have a national screening program. However, there are several local/regional programs and activities such as: the public awareness of BC through lectures, in a major hospital in Riyadh (Abulkhairoa et al., 2010), a year-round, well-designed, public awareness program; and the first, organized population-based mammography screening program, in Alqassim region (Ravichandran et al., 2011).

Very little information exists on the perceptions and beliefs of females in Saudi Arabia about BC and BC control. It is well-known that to improve cancer control, it is important to understand what they know about the disease, its screening, early detection and treatment (Dandash and Al-Mohaimeed, 2007). 

In KSA, studies related to knowledge, attitudes, and practices around BC are scarce. Madanat and Merrill (2002) found a very low level of knowledge of BC and its associated risk factors among female high-school students (Irurhe et al., 2011). However, an older female population from Riyadh, was found to be more knowledgeable about BC. Among 864 women aged 20–50, living in Riyadh, 82% knew about breast self-examination, and 61% knew about mammographies. However, only 41.2% had performed breast self-examinations, and only 18.2% had ever had a mammogram (Rojas and Stuckey, 2011). In Al Hassa governorate, a population-based study found even lower rates of mammography (5.1% among 1,315 women aged 18–65 years old) (Elmore et al., 2005). Another study of teachers in their thirties, also showed low levels of breast-cancer-related knowledge, with only 32.4% being aware of breast self-examination (Abulkhair et al., 2010). Whether Saudi women know about BC screening and whether those above 50 years are being screened for BC at least once every two years is unknown. We conducted a regional survey, to investigate knowledge and practices of BC screening among school teachers, in order to assess the need for BC and cancer awareness national health programs.

Several studies have shown inadequate levels of knowledge towards risk factor awareness and cancer screening (like clinical breast examinations and mammogram), even among educated women (Amin et al., 2009).

In Saudi Arabia, a few studies have been carried out to assess awareness towards risk factors of BC, with some methodological limitations (Alam, 2006; Bener et al., 2009). While none exist assessing the distribution of various risk factors implicated in BC, as well as screening behavior. Consequently, the objective of this study was to assess level and determinants of knowledge about, risk factors, behaviorand utilization of screening methods used for BC.

Cost and availability of health insurance have been found to act as barriers to BC screening in the U.S. and other parts of the world, but not in Saudi Arabia, where a mammography is usually either free or covered by insurance (Milaat, 2000; Bener et al., 2009).

We aim in this study to assess knowledge, awareness, attitude and practice of Saudi females towards BC and screening for BC. We picked school teachers as our sample, because they constitute a large number of the population; thus the result of the study, we believe, can be applied to the whole population of Saudi Arabia.

Also, school teachers are classified as a ‘well-educated’ sector in the country and it is expected that they are well aware of and oriented about BC.

Finally, we couldn’t find any similar study done in the Makkah region and we hope that with this study we can attract some attention, to develop awareness and screening programs for BC here.

## Materials and Methods

*Study Design*

*Cross-sectional survey in the city of Makka*h

Study Population and sample size: A total sample of 400 school teachers working in primary, intermediate and secondary schools, were selected by multistage random sampling. Forty schools in the city of Makkah were selected randomly, and a sample of 10 teachers were randomly selected from each school. Sample size was determined using sample size determination tables, as according to the ministry of education’s website, the total number of teachers is 12905 and the sample size is 365. However, considering 10% non response rate the final sample size is 400.

*Instruments*

A questionnaire was developed based on a comprehensive review of literature, to include questions on women’s awareness level about different aspects of BC. The original questionnaire was in English, for which translation into Arabic, with back translation to English was done, to preserve the original construct. Content validity was reviewed by 2 experts in the field. There were twenty-three questions covering the important aspects of BC that the public should know, including: risk factors, signs, screening, treatment methods, screening centers in Makkah and the information sources for each. Questions assessing knowledge score were given a value of one if correct and zero if incorrect. The total knowledge score was 37.

Data collectors were recruitedand trained for 2 days. In the training session, the data collectors were oriented about the subject itself; we give them a workshop to explain objectives of the study anddata collection process. All collected data was reviewed by the supervisors and principle investigators.

Initially, we did a pilot study on 10 school teachers to ensure quality, clarity and completeness of data.

*Statistical Analysis*

Data was analyzed using SPSS software, version 21. Scores of knowledge items were summed to obtain the mean total knowledge score on BC, total scores were found to be normally distributed. Descriptive tests (frequency, mean, standard deviation (SD) and percentages) were done to characterize different variables. Parametric tests (T-test and ANOVA test) were applied to compare knowledge across the socio-demographic variables. Multiple linear regression analysis was performed to obtain the significant predictors of BC knowledge. The level of significance was set below 5% (p<0.05).

*Ethical Consideration*

Participants were informed about the nature of the study. Written consent was obtained. All information obtained from participants was anonymous. Approval from the Research Ethical Committee in the faculty of medicine at Umm Al-Qura University was obtained and the study was conducted after approval.

## Results

The response rate was 100% as all participants responded to the questionnaire; the majority of the respondents were between 36 and 45 years old (49%), married (70%) and held a bachelors degree (77%). Seventy eight percent of them didn’t have a family history of BCand 81% haven’t had any breast disease before Table 1.

Table 2 Illustrates respondents knowledge about risk factors and warning signs. Alcohol was the most identified risk factor (82%), followed by family history (69%) andlong term oral contraceptive pill use (66%). Increased maternal age at first pregnancy (0.9%) was the least identified risk factor, followed by late menopause (hormonal therapy exposure) (3%) and early menarche (5%). The majority of respondents identified a lump under the armpit (75%) as a BC warning sign, followed by a painless breast lump (65%) and bleeding or discharges from the nipple (52%). Weight loss (22%) and nipple pain (35%) were the least identified warning signs of BC.

Respondents’ mean scores of their overall knowledge about BC based on some demographical characteristics: It is found that the overall mean score for the participants’ knowledge levels about BC is 15.6±4.19. Most participants’ (67%) had a weak knowledge score, followed by an average knowledge score of (24%).the lowest percentage of participants’ (6%) had a good knowledge score.

School teachers’ knowledge about BC differed significantly by their age and marital status, as those aged 46-55 (F=8.5, p>0.00) and those who are married (F=2.7, p>0.04) had more knowledge about BC than others .

Table 3,4, Univariate linear regression analysis was done to the age, marital status, education level, those with family history of BCand those who had breast disease. Multi-collinearity was checked and the analysis showed no intercorrelation among the independent variables. Only age was statistically significant (P < 0.05).

Table 5, Forty five percent of participants’ knew about BC Screening centers in Makkah city, 93.3% of respondents think that early BC screening is important, 92.3% stated that they would go to see a doctor only if they had breast problem and over half of the participants would agree to participate in a BC screening program.

Fifty-one participants had undergone a mammography and only 31 out of them were for screening. Out of all participants, 57% thought that BC treatment depends on the stage (Table7).

**Table 1:** Socio-Demographic Characteristics of the Respondents (n=400)

Characteristic	N	%
Age		
25-35	145	36.3
36-45	197	49.3
46-55	58	14.5
Marital Status		
Single	71	17.8
Married	282	70.5
Divorced	32	8
Widowed	15	3.8
Educational Level		
Diploma	66	16.5
Bachelor	311	77.8
Master	15	3.8
Doctorate	2	0.5
Others	6	1.5
Family History of Breast Cancer		
Yes	63	15.8
No	314	78.5
I don’t know	23	5.8
*Who Had Breast Cancer In Family		
Mother	21	33.3
Sister	5	7.9
Ant	14	22.2
Grandmother	5	7.9
Others	18	28.6
Had Breast Disease		
No	227	81.5
breast inflammation	15	3.7
breast ulcer	3	0.7
breast tumor	4	1
nipple secretions	23	5.7
breast lump	27	6.7
other breast problem	2	0.5
Have you ever had a mammogram ?	51	12.8
Yes	51	12.8
If yes why?		
For Screening	36	70.59*
For Diagnosis	15	29.41*
have you ever done breast self-examination?		
Yes	169	42.3
if no why?		
I did not know it excited	37	15.7
I don't know how to do it	120	50.8
I don't think it's important	14	5.9
I don't think I need it	65	27.5

*For participants' who answered Yes (n=63).

**Table 2:** Respondents Knowledge about Risk Factors and Warning Signs of Breast Cancer

Item	True	
	n	%
Risk Factors:		
Family History	278	69.50
Early menarche	23	5.80
Old age of first pregnancy	25	0.90
Early menopause	101	25.30
Aging	104	26.00
Increase maternal age at first pregnancy	25	6.30
Early menopause	101	3.70
Number of pregnancies'	84	21.00
Infertility	68	17.00
Staying Single	46	11.50
Obesity	113	28.30
Fatty foods	134	33.50
OCP use	265	66.30
Increased Stress Levels	275	68.80
X-ray Exposure	315	78.80
Smoking	251	62.80
Alcohol drink	331	82.80
Worming Sings:		
Painless Breast Lump	261	65.40
Changes in the size of breast or nipple	192	48.10
Changes in the shape of breast or nipple	203	50.90
Bleeding or discharge from the nipple	210	52.60
Pulling of the nipple	168	42.10
nipple pain	142	35.60
Wight loss	88	22.10
Redness of the breast skin	171	42.90
Lump under armpit	300	75.20

**Table 3:** Participants' Knowledge Score Toward BC

	Disease knowledge score = 30		Screening knowledge score = 7		Overall knowledge score = 37	
	N	%	N	%	N	%
Weak*	246	61.5	202	50.5	271	67.75
Average*	99	24.7	182	45.5	96	24
Good*	6	1.5	6	1.5	6	1.5
Range	3-24		3-6		6-29	
Mean±SD	12.27±3.93		3,33±1.11		15.6±4.19	
Chi-square	286.8		197.6		205.11	
	<0.001		<0.001		<0.001	

*Weak, (score 49-25%); Average, (score 50-74%); Good, ( score ≤75%)

**Table 4:** Differences in Respondents Breast Cancer Knowledge by Demographic Variables

Characteristic	Mean	SD	F	P-Value
Age				
25-35	17.62	3.608	8.516	0
36-45	18.81	3.902		
46-55	19.98	4.47		
Marital Status				
Single	17.46	3.66	2.78	0.041
Married	18.89	4.044		
Divorced	17.94	3.991		
Widowed	18.47	2.669		
Educational Level				
Diploma	19.05	4.145	0.721	0.578
Bachelor	18.42	3.938		
Master	18.4	3.699		
Doctorate	22	7.071		
Others	18.67	2.944		
Family History of Breast Cancer				
Yes	18.73	4.064	1.545	0.215
No	17.87	3.348		
I don’t know	17.91	3.942		
Had Breast Disease				
Yes	18.48	3.969	-0.162*	-0.083*
No	18.56	3.963		
Practice Breast Self Examination				
Yes	19.07	4.121	2.32*	0.021*
No	18.13	3.756		
Had Mammography				
Yes	19.65	5.047	2.193*	0.085*
No	18.36	3.726		

*T-statistic and p-value are based on the results of t-test.

**Table 5:** Predictors of Breast Cancer Knowledge by Univariat Linear Regression (n=400)

	B	SE	Beta	P value	95% CI	
					Lower	Upper
Age	1.182	0.286	0.203	0	0.62	1.745
Marital Status	0.337	0.31	0.054	0.277	-0.273	0.947
Educational Level	-0.136	0.334	-0.020	0.685	-0.792	0.521
Family History	-0.572	0.353	-0.081	0.106	-1.26	0.122
Had Breast Disease	-0.083	0.513	-0.008	0.871	-1.09	0.926

**Table 6:** Participants' Attitude Toward Screening Programs

Question	n	%
do you know any breast cancer screening center in Makkah?	181	45.3
do you think early breast cancer screening is important?	385	96.3
Would you agree to participate in a Breast Cancer screening Program If offered?	237	59.3
if you or a family member had any breast problem would you go to the doctor?	369	92.3
have you ever had a breast cancer examination by a doctor?	107	26.8

**Table 7:** Participants' Knowledge About Breast Cancer Screening and Treatment

Question	n*	%*
What percentage do you think the risk of breast cancer in women? (<25%)	155	38.8
What is mammogram? (breast x-ray)	33	3.8
How often should mammogram be done? (annually)	93	23.3
At what age do you think mammogram screening should start? (50)	32	8
Have you ever had a mammogram? (yes)	51	12.8
If yes why?		
For Screening	36	70.59*
For Diagnosis	15	29.41*
have you ever done breast self-examination? (yes)	169	42.3
if no why?		
I did not know it excited	37	15.7
I don't know how to do it	120	50.8
I don't think it's important	14	5.9
I don't think I need it	65	27.5
What is the treatment of breast cancer?		
Chemotherapy and radiotherapy	69	17.3
Hormonal therapy	16	4
Surgery or removal of the whole breast	84	21
It depends the stage	231	57.7

* number and percentage of participants who answered correctl

## Discussion

In Saudi Arabia, BC is the most common cancer among females. It usually presents at advanced stages andit is considered the leading cause of cancer mortality in women (14% of female cancer deaths). Since it is known that education and awareness lead to better screening and subsequently early detection (which contribute to better treatment and prognosis), we designed this study toinvestigate the level of BC awareness among Saudi female’s teachers in Makkah. As we can see, only 3.8% know what a mammogram is and only 8% know that it should be started at the age of 50. These results, unfortunately, reflect the poor knowledge of the study group and when we looked at the literature, there was no similar study which asked the same questions. When it comes to the treatment options, we also found that there is relatively poor knowledge about choices of treatment for breast cancer (57% know that it depends on the stage). 

Our findings show that the overall mean score for the participants’ information levels about breast cancer is 15.6±4.19. Most of the participants’ (67%) had a weak knowledge score of 49-25%, four percent had an average knowledge score of 50-74%, and the lowest percentage of participants’ (6%) had a good knowledge score of ≥75%. 

Regarding the screening methods: 50% of our sample had a weak knowledge and 1.5% had a good score.

This finding also supports previous study by Alam et al11 (2006); she reported smoking, hormone replacement therapy and exposure to excess radiation as common BC risk factors noted by women in Riyadh.

Regarding women’s’ awareness of BC warning signs; painless breast lump was the most frequently identified symptom (65%), followed by bleeding or nipple discharge (52%) and change in the shape of breast or nipple (50%). Knowledge of other warning signs were limited, as only few females knew that weight loss (22%) and nipple pain (35%) are warning signs of Radi et al., (2013) reported similar findings in their study. 

This finding is in agreement with what has been reported by Parsa et al., (2008) in their studythey reported that the majority of participants had a low level of knowledge (63%).

When we compared the demographic variables of our population, we noted that marital status and age affected awareness level significantly; as older married females had learned more about the disease and were more aware about screening methods. However, we found that educational level didn’t affect awareness about BC, as more educated females didn’t necessarily know more about the disease or its screening.

The results were consistent with the literature, as published by Dandash and Al-Mohaimeed (2007), and Milaat (2000) on the other hand, these studies showed the significant effect of education on awareness levels, unlike our study, which showed no difference. We think that discrepancy is related to the study group itself. In the last part of the study, we measured the response to screening programs in the literature Radi et al which reported better knowledge in Jeddah city, which is not far from Makkah and has almost similar population demography. We think this difference is mainly due to the society and awareness campaigning, which is usually in Jeddah city.

In conclusions, Brest Cancer is the most common cancer and the second leading cause of deaths among women worldwide. Unfortunately, there is huge defect in the awareness among females in Makkah and in the country in general.
